# Comparison of the Immunomodulatory Properties of Three Probiotic Strains of *Lactobacilli* Using Complex Culture Systems: Prediction for In Vivo Efficacy

**DOI:** 10.1371/journal.pone.0007056

**Published:** 2009-09-16

**Authors:** Erika Mileti, Gianluca Matteoli, Iliyan D. Iliev, Maria Rescigno

**Affiliations:** Department of Experimental Oncology, European Institute of Oncology, Milan, Italy; University of Toronto, Canada

## Abstract

**Background:**

While the use of probiotics to treat or prevent inflammatory bowel disease (IBD) has been proposed, to this point the clinical benefits have been limited. In this report we analyzed the immunological activity of three strains of *Lactobacillus* to predict their in vivo efficacy in protecting against experimental colitis.

**Methodology/Principal Findings:**

We compared the immunological properties of *Lactobacillus plantarum* NCIMB8826, L. *rhamnosus* GG (*LGG*), *L. paracasei* B21060 and pathogenic *Salmonella typhimurium* (SL1344). We studied the stimulatory effects of these different strains upon dendritic cells (DCs) either directly by co-culture or indirectly via conditioning of an epithelial intermediary. Furthermore, we characterized the effects of these strains in vivo using a Dextran sulphate sodium (DSS) model of colitis.

We found that the three strains exhibited different abilities to induce inflammatory cytokine production by DCs with *L. plantarum* being the most effective followed by *LGG* and *L. paracasei*. *L. paracasei* minimally induced the release of cytokines, while it also inhibited the potential of DCs to both produce inflammatory cytokines (IL-12 and TNF-α) and to drive Th1 T cells in response to *Salmonella*. This effect on DCs was found under both direct and indirect stimulatory conditions – i.e. mediated by epithelial cells - and was dependent upon an as yet unidentified soluble mediator. When tested in vivo, *L. plantarum* and *LGG* exacerbated the development of DSS-induced colitis and caused the death of treated mice, while, conversely *L. paracasei* was protective.

**Conclusions:**

We describe a new property of probiotics to either directly or indirectly inhibit DC activation by inflammatory bacteria. Moreover, some immunostimulatory probiotics not only failed to protect against colitis, they actually amplified the disease progression. In conclusion, caution must be exercised when choosing a probiotic strain to treat IBD.

## Introduction

The intestine is home to trillions of commensal bacteria that participate in digestive functions while helping to protect the host from the aggression of pathogens [1]. Commensals are not ignored by the immune system, rather they are tolerated via a concerted action of epithelial cells and immune cells [2]–[4]. The interaction of commensals with pattern recognition receptors on the apical surface of epithelial cells is protective against colitis [5]–[8], but, commensals can also be colitogenic as several susceptible mice when reared under germ free conditions do not develop colitis [9].

Given this dual role of bacteria – protective versus colitogenic – the use of probiotics as therapeutic agents in IBD has been proposed [10]–[12]. Probiotics can be considered as those microorganisms that are beneficial to the host. According to an FAO/WHO joint report ‘there is good evidence that specific strains of probiotics are safe for human use and able to confer some health benefits on the host, but these benefits cannot be extrapolated to other strains without experimentation’ [13]. Indeed, given the heterogeneity of probiotics and the two-faced character of bacteria as colitogenic or protective, it is clear that not all microorganisms may have the same effect on the host. Instead, it seems likely that each probiotic species, and within each species each strain, may have distinct activities. In agreement with this, the therapeutic effect of probiotics in patients with IBD depends on the strain of probiotic that is used, on the stage of the disease, and on the analyzed pathology (reviewed in [12]). Hence the correct use of probiotics as therapeutic agents calls for a precise knowledge of their activity.

Table 1 encapsulates the previously published studies detailing the activity of different probiotics in protecting animals against experimental colitis. From this it can be appreciated that the activity of certain strains depends on the colitis model used. For instance, in rats, *Lactobacillus* GG (from now on called LGG) has been shown to have no protective effect against dinitrobenzene sulfonic acid (DNBS)- or trinitrobenzenesulfonic acid (TNBS)-induced colitis [14], [15], a partially protective effect in the iodoacetamide colitis model [15], and a detrimental effect in the DSS model [16]. It is also interesting to note that within the same species, different strains can behave differently. For instance, in mice, *Lactobacillus plantarum* DSM 15313 [17] has been shown to partially protect against DSS colitis while the strains ACA-DC 287 [18] or Lp115 [19] have no effect in TNBS colitis.

**Table 1:** Summary of the effect of different probiotics on animal models of colitis.

Probiotic	Colitis model	Protective effect	Presumed mechanism of action	Reference
Lacidofil	Rats: TNBS+Candida	Yes	Reduced colonic damage, reduced serum inflammatory cytokines	[51]
Bacillus subtilis PB6	Rats: TNBS	Yes	Reduced colonic damage, reduced serum inflammatory cytokines	[52]
VSL#3	Mice: DSS	Yes	Prevented the DSS-induced increase of permeability and apoptosis	[23]
E. feacalis, or L. acidophilus, or C. butyricum or B. adolescentis	Mice: DSS	Yes, E. Faecalis better effect	Reduced DAI and MPO activity.	[53]
Mixture of four Lactobacillus or four Bifidobacterium species	Mice: DSS	Yes	Reduced weight loss, colonic damage, inflammatory cytokine expression in colon.	[54]
F. prausnitzii	Mice: TNBS	Yes	Antinflammatory effect: able to block NF-kappaB activation and IL-8 production on Caco-2 cells.	[20]
			Reduction of colonic inflammatory cytokines.	
LGG or mixture of S. thermophilus, L. acidophilus, and B. longum	Rats: TNBS	No	Reduction of pathogenic species. Only LGG reduced MPO activity but not statistically significant.	[14]
VSL#3	Mice: IL-10 KO	Yes	Reduced inflammation and upregulation of mucosal alkaline sphingomyelinase activity	[55]
E. coli M-17	Mice: DSS	Yes	Reduced IL-1beta, reduced severity of disease	[56]
L. plantarum DSM 15313, L. fermentum 35D.	Mice: DSS	Yes	Improved the DAI, reduced bacterial translocation, and reduced inflammation	[17]
L. salivarius 433118	Mice: DSS IL-10 KO	No effect		[57]
L. fermentum ACA-DC 179, L. plantarum ACA-DC 287 and S. macedonicus ACA-DC 198	Mice: TNBS	No effect of L. plantarum and S. macedonicus. Protection by L. fermentum.	Reduced inflammation by unknown mechanism	[18]
L. casei	Mice: TLR-4 KO and DSS	Yes	Reduced colitis and reduced expression of inflammatory cytokines. Down-regulation of neutrophil recruitment	[21]
L. casei, L.acidophilus and B. lactis	Rat: TNBS	Yes	Intestinal anti-inflammatory effects, significant reduction in the colonic weight/length ratio	[58]
L.s gasseri expressing MnSOD	Mice: IL-10 KO	Yes	L. gasseri producing MnSOD significantly reduced inflammation and infiltration of neutrophils and macrophages	[22]
Several strains	Mice:TNBS	Yes	Correlation between high IL-10/IL-12 and protective effects against colitis	[44]
L. reuteri and L. fermentum	Rats: TNBS	Yes: L. fermentum more effective.	Both strains reduced TNFalpha levels and inducible NO synthase	[59]
LGG, S. thermophilus TH-4, B. lactis Bb12 and L. fermentum BR11	Rats: DSS	Yes and no: differences among the strains	B. lactis Bb12 and L. fermentum BR11 decreased severity of colitis.	[16]
			TH-4 did not prevent DSS-colitis and LGG actually exacerbated the disease.	
L. salivarius Ls-33; L. plantarum Lp-115 and L. acidophilus NCFM	Mice: TNBS	Yes and no: differences among the strains	L. salivarius Ls-33 had a preventive effect on colitis in mice. Lactobacillus plantarum Lp-115 and L. acidophilus NCFM had no effect. L. paracasei exacerbated colitis	[19]
E. coli Nissle 1917	Mice:TLR-2, TLR-4 KO+DSS	Yes	E. coli Nissle ameliorated colitis only in WT colitic mice, but not in the two KO suggesting TLR-2 and TLR-4 dependent mechanisms.	[60]
L. delbrueckii bulgaricus B3 or L. delbrueckii bulgaricus A13	Rats: Acetic acid	Yes	Reduced colonic damage and myeloperoxidase activity in both probiotic-treated groups, but high-EPS group even better scores.	[61]
VSL#3	Mice: DSS	No	No effect on colitis or on epithelial barrier repair	[62]
L. casei Shirota	Mice: DSS	Yes	Reduced disease severity	[63]
L. fermentum	Rats: TNBS	Yes	Amelioration of the inflammatory response and reduced MPO activity	[64]
L. casei DN-114 001	Rats: TNBS	Yes	Reduced inflammation and reduced barrier disruption.	[65]
L. casei Shirota	Mice: DSS and SAMP1/Yit	Yes	Reduced colitis and reduced IL-6 production by LPMC	[66]
E. coli Nissle1917	Mice: acute DSS; chronic IL-10 KO	Yes	Acute model: Nissle1917 ameliorated body weight loss, DAI	[67]
			Chronic model, suppressed inflammation and histologic damages	
VSL#3	Mice: TNBS (recurrent colitis)	Yes	Increased production of IL-10 and number of regulatory CD4+ T cells bearing surface TGF-beta	[24]
L. crispatus M247	Mice: DSS	Yes	Reduced the severity of colitis (but only the aggregating form of bacteria)	[68]
E. coli Nissle 1917	Mice: Acute: DSS; chronic: T cell transfer in SCID mice	Yes	Ameliorated acute and chronic experimental colitis by reduced inflamamtion	[69]
L. salivarius 433118 and B. infantis 35624	Mice: IL-10 KO	Yes	Reduce colonic and caecal inflammatory scores. Reduced inflammatory cytokines. TGF-b levels maintained.	[70]
VSL#3 or LGG	Rats: DNBS; iodoacetamide	Yes and no depending on colitis model. Decreased inflammation in iodoacetamide model. No effect on DNBS.	Reduced inflammation but decrease in PGE2, MPO, and NOS activity.	[15]
L. plantarum 299v	Mice: IL-10 KO	Yes	Unknown	[71]
Lactobacillus and C. butyricum	Rats: DSS	No effect		[72]
L. salivarius UCC118	Mice: IL-10 KO	Yes	Reduced mucosal inflammatory activity and cancer	[73]
L. plantarum 299	Rats: TNBS/E	No effect		[74]
IL-10-secreting Lactococcus lactis	Mice: DSS; IL-10 KO	Yes	IL-10 mediated effect	[75]

*Abbreviations used:*

Disease activity index (DAI).

Myeloperoxidase activity (MPO).

Manganese superoxide dismutase (MnSOD).

Inducible nitric oxide synthase (iNOS).

Exopolysaccharide (EPS).

Lamina propria mononuclear cells (LPMC).

dinitrobenzene sulfonic acid (DNBS).

trinitrobenzenesulfonic acid/ethanol (TNBS/E).

The mechanisms of action of probiotics can be quite disparate. They have been shown to modulate the permeability of epithelial barriers, alter the inflammatory potential of epithelial cells, compete with pathogens for mucosal colonization, or directly modify the activity of immune cells (reviewed in [10], [11]). However, the principal mechanism of protection against colitis is via a reduction in the release of pro-inflammatory cytokines, but the pathways and cells involved in this mechanism are not yet clear. Some probiotics like *Faecalibacterium prausnitzii* (whose reduction in the normal gut microbiota has been associated with Crohn's disease (CD) recurrence [20]) can inhibit NF-κB activation and IL-8 production by Caco-2 cells and reduce colonic inflammatory cytokines in colitic mice [20]. In other cases, probiotics like *Lactobacillus casei* or *Lactobacillus gasseri* expressing a manganese superoxide dismutase (MnSOD) can reduce inflammation via the inhibition of neutrophil recruitment [21], [22]. Another mechanism of action of probiotics has been described for a mixture of eight probiotics named VSL#3 [23] or for *Lactobacillus plantarum* DSM 15313 [17], which relies upon an attenuation of the increased epithelial barrier permeability caused by DSS and the resulting bacterial translocation. In addition, VSL#3 can induce the differentiation of protective T regulatory cells [24]. This activity of probiotics may be exerted directly on dendritic cells (DCs), which are professional antigen presenting cells, as probiotic-loaded DCs can modulate the TNBS-induced inflammatory reaction through the differentiation of protective T regulatory cells [25].

DCs are pivotal in the initiation of adaptive immune responses and can directly contact and internalize intestinal bacteria [26]–[28]. Further, DCs can receive tissue conditioning by intestinal epithelial cells that control the DC inflammatory potential [29]–[31]. Hence, probiotics can interact either directly with DCs or indirectly, via the action of epithelial cells. In this manuscript we used a technique established in our laboratory to evaluate the ability of three *Lactobacilli* strains (*plantarum*, *LGG* and *paracasei* B21060) to activate DCs either directly or indirectly via the action of epithelial cells. *LGG* and *L. plantarum* were chosen in order to compare already published reports while *L. paracasei* has been chosen for its capacity to survive the gastrointestinal tract, temporarily associate with the intestinal wall in humans [32], [33] and inhibit T cell proliferation [34]. We found that the activity of the three probiotics was very different. *L. paracasei* the more immunomodulatory among the three strains, was able to inhibit the inflammatory potential of pathogenic *Salmonella* and protect against experimental colitis.

## Results

### Probiotics induce the phenotypical maturation of DCs

**Figure 1:**
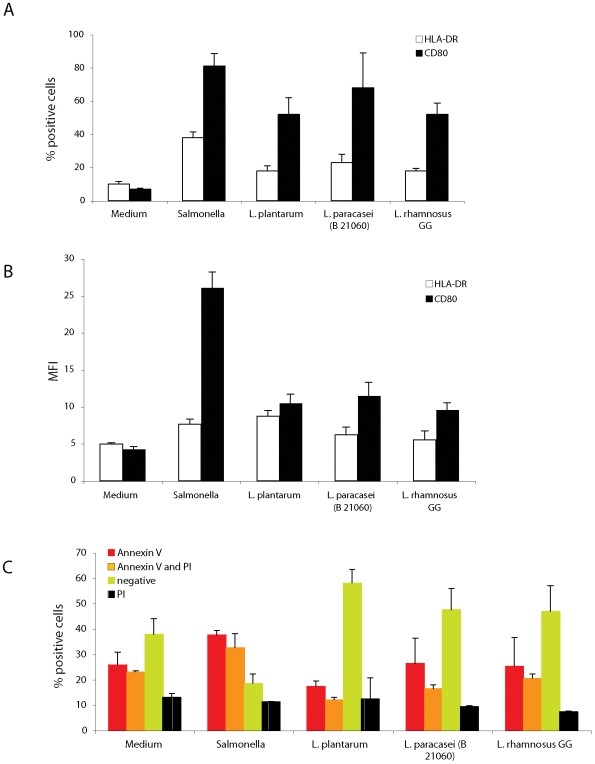
DCs are phenotypically similarly activated by *Lactobacilli*. DCs were incubated or not with the reported live bacterial strains for 1 h in medium without antibiotics, washed and incubated for 23 h in medium with antibiotics. Cells were stained for HLA-DR and CD80 expression and analyzed by FACS. A. % of cells highly positive for the marker is reported. B. Mean fluorescence intensity (MFI) expression of markers is reported. C. Viability of the cells after 24 h incubation with bacteria. Cells were stained with propidium iodide (PI) and annexin V and analyzed by FACS. Cells double negative for both markers are considered viable cells. Error bars: standard deviations on values obtained on 4 different donors.

The first outcome of the encounter of DCs with bacteria is the upregulation of surface activation markers. We generated DCs from human peripheral blood monocytes (MoDCs) of 4 different donors to assess donor-associated variability. MoDCs were incubated for 1 h with live logarithmic-phase *Lactobacilli* (*L. plantarum*, *L. paracasei* and *LGG*) or with *Salmonella typhimurium* in medium without antibiotics at a 10∶1 (bacteria∶DC) ratio. Cells were extensively washed and the medium was changed to one containing antibiotics. Cells were tested 24 hours later for upregulation of MHC II (HLA-DR) and costimulatory (CD80) molecules. Consistent with a recent report on bone marrow-derived DCs [35], the three probiotic strains induced a similar upregulation of HLA-DR and CD80, which was however reduced as compared to the upregulation induced by *Salmonella* (Fig. 1A-B, Figure S1). Given the low multiplicity of infection and the short exposure time, the viability of the cells (as seen by the frequency of propidium iodide (PI) negative and annexin V negative cells), was similar after each probiotic treatment, while *Salmonella* was partially toxic (Fig. 1C).

### Probiotics have a different ability to induce cytokine production by DCs

**Figure 2:**
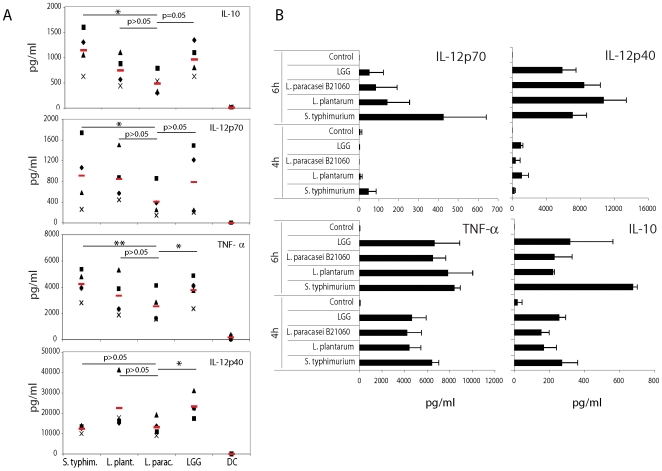
DCs incubated with different bacterial strains produce a distinct cytokine profile. A. DCs were incubated or not with the reported live bacterial strains for 1 h in medium without antibiotics, washed and incubated for 23 h in medium with antibiotics. Culture supernatants were collected and tested for cytokine contents by ELISA. Each symbol represents a different DC donor. Red lines represent mean values. *, p<0.05; **, p<0.01. S. typhim.: *S. typhimurium*; L. plant.: L. plantarum; L. parac.: *L. paracasei*. B. To analyze the kinetic of cytokine production, DCs were incubated or not with the reported live bacterial strains for 1 h in medium without antibiotics, washed and incubated for 3–5 h in medium with antibiotics. Culture supernatants were collected and tested for cytokine release by ELISA. Error bars: standard deviations on values obtained on 4 different donors.

Phenotypical activation of DCs does not necessarily correlate with their functional activation [36] and the type of cytokines released can have an impact on T cell polarization. Therefore, we analyzed the production of IL-12p70, IL-10, TNF-α and IL-12p40 by MoDCs after 24 h treatment with bacteria, as above. *Salmonella* was a strong inducer of all of the tested cytokines, while the three *Lactobacilli* elicited differential cytokine release (Fig. 2A). *L. plantarum* and *LGG* induced a cytokine response that was very similar to that of *Salmonella*, while *L. paracasei* induced lower levels of IL-12p70, TNF- α and IL-10 when compared to *Salmonella*. Thus, the only strain displaying a reduced inflammatory potential was *L. paracasei*.

It has been shown that IL-10 can negatively regulate the expression of IL-12p70 [37]. To test whether an early increase in IL-10 release could impact on IL-12 production, we analyzed IL-10 release during the initial phases of DC activation (4–6 hours). The levels of IL-10 were not higher in any of the cultures of DCs with *Lactobacilli* during the times at which IL-12p70 was low, thereby suggesting IL-12p70 induction is delayed in comparison to *Salmonella* and is not controlled by IL-10 (Fig. 2B).

### The difference in cytokine production reflects different T cell polarizing ability

**Figure 3:**
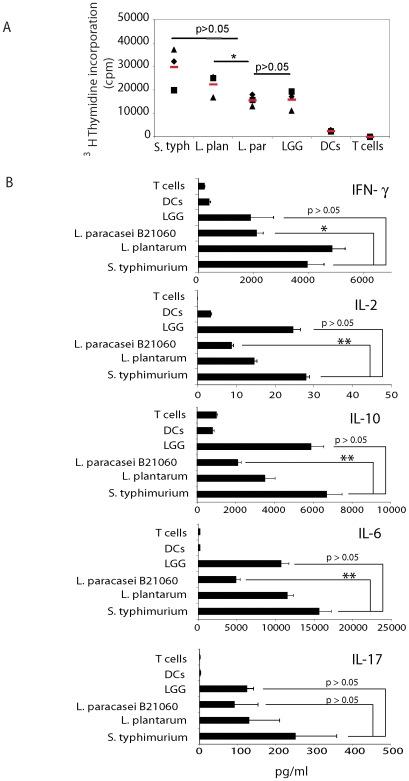
*Lactobacilli*-treated DCs have different ability to induce T cell proliferation and cytokine production. A. T cell proliferation: DCs were incubated or not with the reported live bacterial strains for 1 h in medium without antibiotics, washed and incubated for 23 h in medium with antibiotics. Bacteria-treated DCs were washed and incubated with naïve CD4^+^CD45RA^+^ cells for 3 days, followed by a 16-hours pulse with 1 µCi [^3^H] thymidine (Amersham, Milan). ^3^H-thymidine incorporation is shown. Each symbol represents a different DC donor. Red lines represent mean values. *, p<0.05. S. typh: *S. typhimurium*; L. plan: L. plantarum; L. par: *L. paracasei*. B. Cytokine release: Bacteria-treated DCs were incubated with naïve CD4^+^CD45RA^+^ cells for 5 days (Ratio 1∶10 DC∶T cells). Cell culture supernatants were collected and cytokines measured by ELISA or CBA Flex set. Error bars: standard deviation on values obtained on 3 different donors. *, p<0.05; **, p<0.01.

Cytokine release by DCs is important to drive the polarization of T cells towards Th1, Th2, Th17 or T regulatory cells. Given the differences observed in cytokine production we analyzed the capacity of bacteria-treated DCs to activate and polarize T cells. DCs were incubated with live bacteria and then cultured with highly purified allogeneic naïve CD4^+^CD45RA^+^ T cells. As shown in Figure 3A, all three *Lactobacilli* were less potent in inducing T cell proliferation when compared to *Salmonella*, probably reflecting their reduced ability to upregulate surface activation markers (Fig. 1A–B). When we analyzed the cytokines produced by T cells we found that T cells activated with *paracasei*-treated DCs were affected in their ability to release IFN-γ, IL-2, IL-10 and IL-6 (Fig. 3B). In contrast, *L. plantarum*-treated DCs activated T cells similarly to *S. typhimurium*-treated cells in terms of IFN-γ release but induced less IL-10, while *LGG*-treated DCs induced the opposite, more IL-10 and less IFN- γ (Fig. 3B). There was no difference in IL-17 production (p>0.05). Some probiotics (i.e. *L. casei* and *reuteri*) can induce the development of T regulatory cells [24], [25], [38]. However, the different *Lactobacilli* and *Salmonella* displayed a similar ability to drive CD25^+^Foxp3^+^ T regulatory cells (Figure S2).

### *L. paracasei* is less inflammatory on epithelial cells

**Figure 4:**
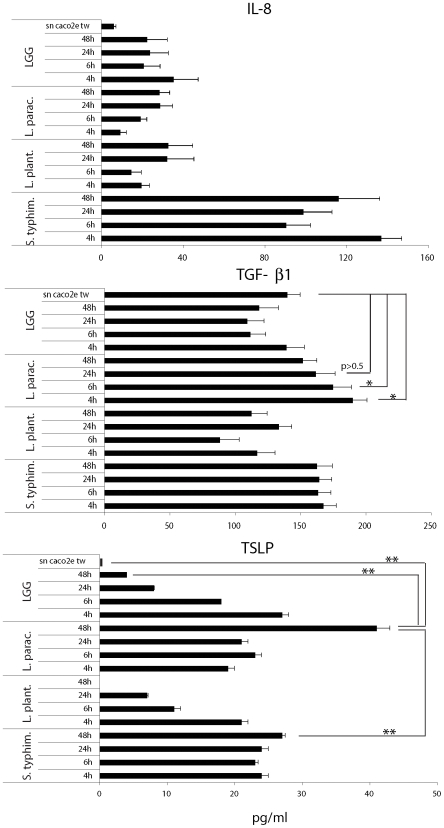
*L. paracasei* induces increased levels of TGF-β and TSLP. Caco-2 cells were grown as monolayers in the upper chamber of a transwell filter and incubated with live bacteria (5×10^7^ CFU/TW) upon the apical surface (top chamber). One hour after incubation, bacteria were washed out and medium was changed with one containing antibiotics. Culture supernatants were collected 3, 5, 23 and 47 hours later from the bottom chamber and tested for cytokine release. Error bars: standard deviations on values obtained in 2 different experiments. *, p<0.05; **, p<0.01.

It is likely that the first cells interacting with the intestinal flora are the epithelial cells (ECs) that line the intestinal wall, especially in IBD patients which display a reduced mucous layer [39]. For this reason, we studied the response of monolayers of ECs to probiotics (5×10^7^ CFU/TW) incubated from the apical (luminal) side. One hour after incubation, bacteria were washed out and medium was changed with one containing antibiotics (gentamycin 100 µg/mL). The different bacteria had no effect on the viability of the cells (not shown). We analyzed the release of IL-8, a chemokine involved in the recruitment of neutrophils, and of thymic stromal lymphopoietin (TSLP) and TGF-β, cytokines shown to inhibit the inflammatory potential of DCs [30], [31]. We found that while *Salmonella* induced the release of IL-8 as early as 4 hours after bacterial treatment, all tested *Lactobacilli* induced negligible IL-8 release (Fig. 4). This probably reflects their inability to invade epithelial cells. *L. paracasei* displayed a greater ability to induce the release TGF-β whose levels were statistically higher than basal levels only at 4 and 6 h post incubation (Fig. 4; p<0.05).

In addition, *L. paracasei* induced TSLP expression that peaked at 48 h from infection, while both *L. plantarum* and *LGG* induced a very transient production of TSLP. *Salmonella* induced sustained TSLP release that did not reach the level of *L. paracasei* (Fig. 4). Interestingly, TSLP mRNA levels were sustained in ECs treated with all bacteria (Figure S3), but the meaning of this observation remains to be understood. One could speculate that TSLP protein is either not released at later time points, or is easily degraded in response to *LGG* and *L. plantarum*. By contrast, *L. paracasei* and *Salmonella* incubation may lead to increased release and/or stabilization of the protein.

### *L. paracasei* inhibits the inflammatory potential of DCs

**Figure 5:**
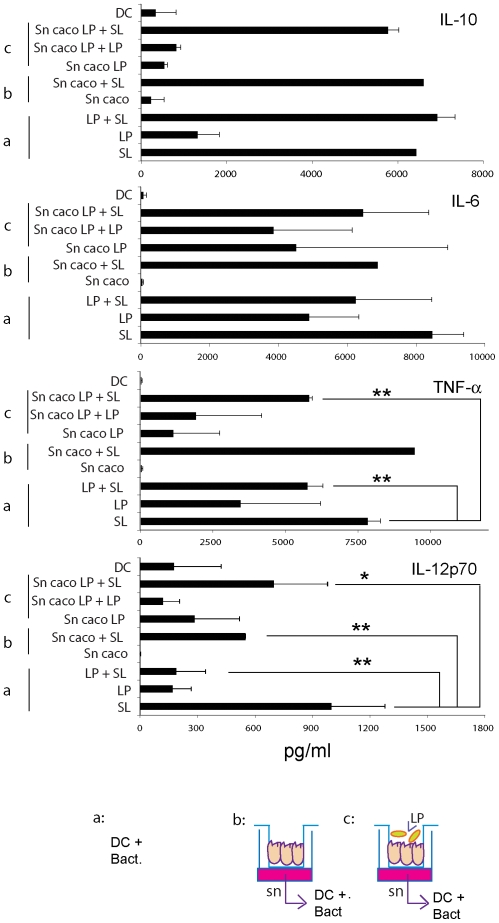
*L. paracasei* inhibits the release of inflammatory cytokines both directly and indirectly on DCs. Each treatment is schematically reported below the graphs. Three situations were analyzed (a, b, c). a. DCs were incubated or not with the reported live bacterial strains either separately (SL, *Salmonella*; LP, *L. paracasei*) or together (LP+SL) for 1 h in medium without antibiotics, washed and incubated for 23 h in medium with antibiotics. b. Caco-2 cells were grown as monolayers in the upper chamber of a transwell filter. 24 h from achievement of a TER of 300 Ohm•cm^2^ supernatants (sn Caco) were collected from the bottom chamber and used to pre-treat DCs for 24 h before bacterial incubation as in a. c. Caco-2 cells were grown as monolayers in the upper chamber of a transwell filter and incubated with *L. paracasei* (5×10^7^ CFU/TW) upon the apical surface (top chamber). One hour after incubation, bacteria were washed out and medium was changed with one containing antibiotics. Culture supernatants (sn caco LP) were collected 24 hours later from the bottom chamber, filtered and used to pre-treat DCs for 24 h before bacterial incubation as in a. 24 h after bacterial treatment of DCs cell culture supernatants were collected and cytokines analyzed by ELISA. Error bars: standard deviations on values obtained on 3 different donors. *, p<0.05; **, p<0.01

Having shown that *L. paracasei* was the least inflammatory among the three *Lactobacilli* strains, we focused on this strain for further experiments. We utilized three different conditions involving the interaction between bacteria, epithelial cells and DCs. DCs were either incubated with: a. *L. paracasei* (LP) and *Salmonella* (SL) individually or together; b. EC supernatant for 24 h and then subsequently with each bacteria; c. supernatants of ECs pre-incubated for 24 h with *L. paracasei* (Sn caco LP) on the apical side and then (24 h later) with each bacterial strain. As shown in Fig. 5, *L. paracasei* had a strong anti-inflammatory effect on DCs both when directly co-incubated with *Salmonella* and indirectly when supernatants of LP-treated ECs were incubated with DCs before exposure to *Salmonella*. The co-incubation of DCs with LP and *Salmonella* significantly reduced the ability of *Salmonella* to induce IL-12p70 and TNF-α, while not altering its ability to promote IL-10 and IL-6 production (Fig. 5). A similar scenario was observed when DCs were first incubated with supernatants of LP-treated ECs and then infected with *Salmonella*. However, as we have already described [29], the incubation of DCs with unconditioned EC supernatant also reduced the ability of DCs to release IL-12p70 but not TNF in response to *Salmonella*. Therefore, the exposure of ECs to LP strongly inhibited the inflammatory response of *Salmonella* on DCs by inhibiting both IL-12p70 and TNF-α release (Fig. 5). This effect may be mediated either by LP-induced release of anti-inflammatory mediator/s by ECs, or by some component of *L. paracasei* that is translocated across the monolayer. The involvement of whole LP translocated across the monolayer is unlikely, as we could not detect intact bacteria from the basolateral side (not shown) and supernatants were filtered before incubation with the DCs. We could not detect any effect of LP on IL-6 or IL-10 (Fig. 5).

**Figure 6:**
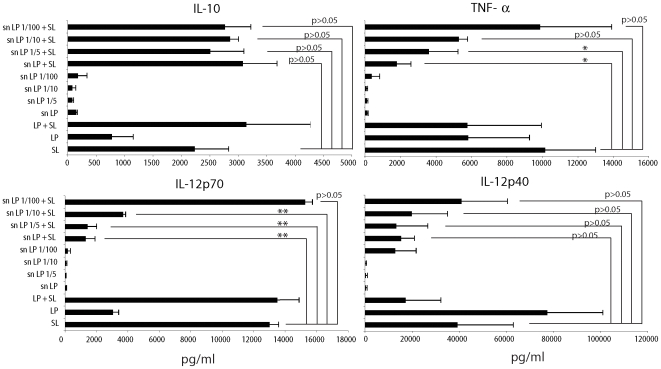
*L. paracasei* culture supernatant is responsible for the anti-inflamamtory activity of the bacterium. DCs were incubated or not with the reported live bacterial strains either separately (SL, *Salmonella*; LP, *L. paracasei*) or together (LP+SL) or in the presence of culture supernatants of *L. paracasei* corresponding to the exponential growth of the same amount of CFU of bacteria used to treat the DCs. The culture supernatant (sn LP) was used either undiluted or diluted 1/5, 1/10, 1/100 that correspond to nearly 7%, 1,4%, 0,7%, and 0,07% volume/volume of tissue culture medium, respectively. Cells were incubated with the different treatments for 1 h in medium without antibiotics, washed and incubated for 23 h in medium with antibiotics. Cytokine release was analyzed by ELISA. Error bars: standard deviations on values obtained on 3 different donors. *, p<0.05; **, p<0.01

We then analyzed whether the factor(s) involved in the anti-inflammatory effect was a soluble mediator and could be found in the culture supernatant of *L. paracasei*. DCs were coincubated with *Salmonella* and either *L. paracasei* or its culture supernatant (sn LP: derived from the same amount of CFUs used for DC incubation). Interestingly, the LP supernatant alone (7% volume/volume of tissue culture medium) was extremely efficient in inhibiting the DC release of inflammatory cytokines while it was unable to alter the ability of DCs to release IL-10 or IL-12p40 (Fig. 6). When LP was extensively washed before incubation with DCs it lost the ability to inhibit the DC release of inflammatory cytokines in response to *Salmonella* (Fig. 6), suggesting that the anti-inflammatory effect of *L. paracasei* is dependent on a soluble metabolite or mediator. It is likely that this mediator(s) is not released during the limited time of LP in culture with the DCs as we could not detect LP growth during the 1 h incubation time with DCs (most likely due to the aerobic culture conditions, not shown). Further diluting the LP culture supernatant 1 to 5 (1.4% volume/volume) but also 1 to 10 (0.7% volume/volume) was still able to inhibit the release of IL-12p70 and TNF-α, indicating the high efficacy of the soluble mediator(s) (Fig. 6).

### Coincubation of *Salmonella* and *L. paracasei* affects the ability of DCs to activate Th1 T cells

**Figure 7:**
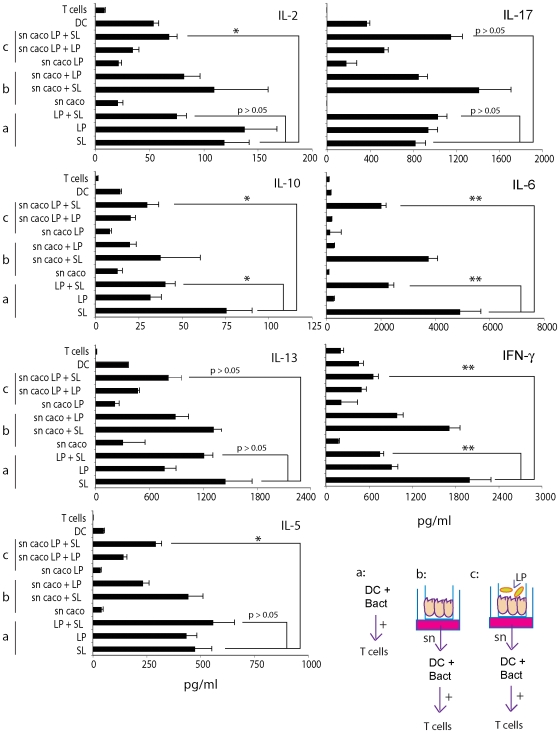
*L. paracasei* inhibits the ability of DCs to activate T cells. Three situations were analyzed (a, b, c) as in fig. 6 DCs were incubated or not with the reported live bacterial strains either separately (SL, *Salmonella*; LP, *L. paracasei*) or together (LP+SL) for 1 h in medium without antibiotics, washed and incubated for 23 h in medium with antibiotics. a. Caco-2 cells were grown as monolayers in the upper chamber of a transwell filter. 24 h from achievement of a TER of 300 Ohm•cm^2^ supernatants (sn Caco) were collected from the bottom chamber and used to pre-treat DCs for 24 h before bacterial incubation as in a. b. Caco-2 cells were grown as monolayers in the upper chamber of a transwell filter and incubated with *L. paracasei* (5×10^7^ CFU/TW) upon the apical surface (top chamber). One hour after incubation, bacteria were washed out and medium was changed with one containing antibiotics. Culture supernatants (sn caco LP) were collected 24 hours later from the bottom chamber, filtered and used to pre-treat DCs for 24 h before bacterial incubation as in a. Cells were then washed and incubated with naïve CD4+CD45RA+ cells for 5 days (Ratio 1∶10 DC∶T cells). Cell culture supernatants were collected and cytokines measured by ELISA or CBA Flex set. Error bars: standard deviations on values obtained on 3 different donors. *, p<0.05; **, p<0.01.

Having shown that the coincubation of DCs with LP and *Salmonella* (SL) drastically reduced the ability of DCs to release IL-12p70 while preserving IL-10 production we analyzed the ability of these DCs to polarize inflammatory T cells. We treated DCs with either LP, or SL or the two together. Cells were then incubated with highly purified naïve T cells and cytokine release in culture supernatants was tested. As shown in Figure 7 (see the situation a), T cells activated with DCs that were incubated with both *Salmonella* and *L. paracasei* were highly impaired in their ability to release IL-2, IL-10, IL-6 and IFN-γ. We could not observe any difference in IL-17, IL-13 or IL-5 suggesting that LP+SL treated DCs were still capable of inducing Th17 or Th2 polarization (Fig. 7).

### DCs incubated with supernatants of *L. paracasei* treated ECs are affected in their ability to drive Th1 T cells

DCs incubated with supernatants of LP-treated ECs are affected in their ability to release IL12-p70 and TNF-α in response to *Salmonella*. Consequently, we evaluated whether this had an impact on Th1 T cell polarization. To accomplish this, DCs were pre-incubated with supernatants from either untreated (sn caco: situation b) or *paracasei*-treated ECs (sn caco LP: situation c) for 24 h, and then with either LP or *Salmonella* for an additional 24 h before incubation with naïve T cells for 5 days. As shown in Fig. 7, the preincubation of DCs with LP-treated-EC supernatants prior to *Salmonella* infection, drastically reduced the DC's ability to activate T cells and drive their polarization to Th1 T cells as evidenced by a decrease in IFN-γ, IL-2 and IL-6 production. There was no difference in IL-17 and IL-13 production (p>0.05) while IL-10 and IL-5 levels were also reduced in culture supernatants (Fig. 7). This indicates that the incubation of ECs with *L. paracasei* has a strong effect on the ability of DCs to activate T cells in response to *Salmonella*.

### The in vitro activity of probiotics predicts their efficacy in vivo

**Figure 8:**
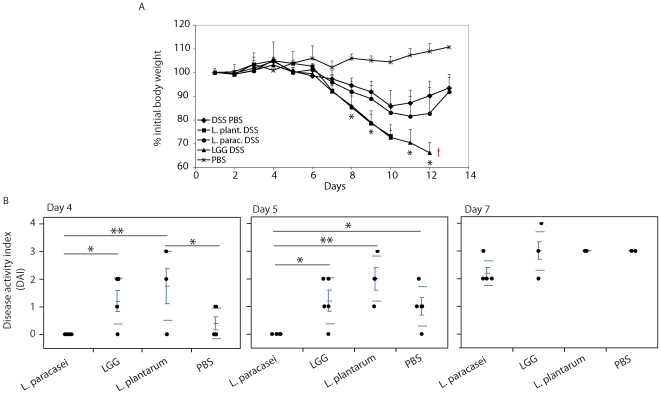
*L. paracasei* protects against DSS colitis. Mice (n = 6) were administered intra gastrically (i.g.) once a day for 7 days with 200 µl PBS containing 10^10^ CFUs of bacteria or plain PBS as a control. Mice were then fed with 2% DSS dissolved in the drinking water for 5 days without bacteria, followed by 7 days of plain water and assessed over time for colitis development. A. Body weight was measured at baseline and every day for the duration of the experiment. Weight change was calculated as percentage change in weight compared with baseline. L. plant.: L. plantarum; L. parac.: *L. paracasei*. Asterisks refer to statistical analysis of the groups LGG or *L. plantarum* versus DSS PBS positive control group. *, p<0.05; **, p<0.01;  = , dead animals. B. Disease activity index (DAI) was measured as reported in Materials and Methods. DAI at 4, 5, 7 days is shown per each group. The scatterplot shows a line at the mean of each group with error bars. Dashed lines identify one standard deviation above and below the group means. *, p<0.05; **, p<0.01.

We next compared the activity of the three *Lactobacilli* in protecting mice against an acute model of colitis. We chose the DSS colitis model as it provokes a strong inflammatory response that is primarily mediated by DCs [40]. Mice were pretreated i.g. for 7 days with 10^10^ CFUs of either *L. plantarum*, *LGG* or *L. paracasei*, or with PBS as a control. Then mice received for 5 days 2% DSS in the drinking water and the development of colitis was followed over time by measurement of body weight, stool consistency and presence of blood in the feces. We found that *L. plantarum* and *LGG*, consistent with their ability to strongly activate DCs, were not only ineffective in protecting against colitis, but were in fact detrimental. Indeed, *LGG*- and *L. plantarum*-treated mice displayed an increased disease activity index (DAI) and all died between 10 and 12 days from DSS administration (Fig. 8). In contrast, mice receiving *L. paracasei* although displaying a similar weight loss as PBS-DSS treated controls, showed a delay in colitis development and a reduced severity of disease (as shown by reduced DAI in Fig. 8B).

## Discussion

IBD is a multifactorial disorder caused by both genetic and environmental factors. The host microflora contributes to disease development and perpetuation as colitis-susceptible mice reared under germ free conditions do not develop the disease and IBD patients treated with antibiotics experience amelioration of symptoms. Further, a colitogenic flora able to disseminate the inflammatory condition has been identified in mice [41]. Finally, it has been shown that a reduced proportion of mucosal associated *Faecalibacterium prausnitzii* is associated with a higher risk of postoperative recurrence of ileal CD [20]. Hence, a lot of effort has been undertaken to find ways to reestablish a protective intestinal flora. In this context, the use of probiotics as therapeutic or preventive agents in IBD has long been proposed, with however limited clinical efficacy [12]. Here we employed both complex in vitro culture systems and an in vivo mouse model to study the immunological effect and anti-inflammatory properties of three different probiotic strains: *L. plantarum*, *LGG* and *L. paracasei*. We show that *Lactobacillus* strains dramatically differed in their ability to activate DCs and to drive the polarization of T cells. In particular, we found that *L. plantarum* and *LGG* were more similar to *Salmonella* in terms of DC activation and T cell polarization than *L. paracasei*. The latter proved very poor in activating DCs and in inducing cytokine production (both inflammatory and non-inflammatory). Furthermore, all *Lactobacilli* induced the development of CD25+Foxp3+ T regulatory cells similarly to *Salmonella*.

When we tested the capacity of the different *Lactobacilli* to activate epithelial cells we found that *L. paracasei* induced an increased release of TGF-β and TSLP. Both factors play an important role in driving the differentiation of non-inflammatory human DCs [30], [31]. Moreover, *L. paracasei* affected the ability of DCs to respond to *Salmonella typhimurium* both directly (when co-incubated with *Salmonella*) and indirectly (after treatment of DCs with supernatants from ECs which had been treated with *L. paracasei* upon their luminal side). Co-incubation of DCs with *Salmonella* and *L. paracasei* together strongly inhibited the release of IL-12p70 while leaving the release of IL-10 untouched. This resulted in reduced differentiation of Th1 T cells. Similarly, the incubation of DCs with supernatants of ECs treated with *L. paracasei* affected the release of IL-12p70 and TNF-α and the development of Th1 T cells. This suggests that once in the intestine, *L. paracasei* may act directly on intraepithelial DCs limiting their ability to induce inflammation even to potent inflammatory bacteria, but they may also indirectly inhibit the activity of DCs that do not enter directly in contact with *paracasei*, via conditioning of ECs. We do not yet know whether this effect is mediated by a soluble factor released by epithelial cells or by a bacterial-derived mediator transcytosed across the epithelial cells. Both possibilities are likely as we found that the direct effect of *L. paracasei* on DCs is indeed dependent on an LP released soluble mediator. The culture supernatant of *L. paracasei* was, by itself, sufficient to inhibit the release of IL-12p70 and TNF-α, but not IL-10, by DCs in response to *Salmonella*. This effect was very strong as it occurred even at very low concentrations of bacterial supernatant (0.7% of the tissue culture volume). Other examples of soluble mediators that are responsible for the anti-inflammatory activity of bacteria have been reported. For instance, *Faecalibacterium prausnitzii* also inhibits IL-8 production induced by IL-1β in Caco-2 cells via an unknown soluble mediator [20]. Similarly, *Lactobacillus acidophilus* exerts its activity via the interaction of the surface (S) layer A protein (SlpA) with the C-type lectin DC-SIGN on dendritic cells [42]. However, it is unlikely that an S layer protein is the mediator of the anti-inflamamtory activity of *L. paracasei* for two reasons. First, *L. paracasei*'s activity is exclusively dependent on a soluble mediator while S layer proteins are also present on the surface of the bacterium. Second, while SlpA alone induces the release of IL-10, as expected through its activation of DC-SIGN, *L. paracasei* supernatant is unable to induce IL-10 production by DCs. Hence, it remains to be analyzed what is the nature of the soluble mediator released by *L. paracasei* and what is the mechanism of inhibition of IL-12p70 and TNF-α release while sparing IL-10 release by DCs.

Therefore, we have described a new property of probiotics, which is to inhibit cytokine release and T cell activation by DCs in response to strong immunogens like *Salmonella*. The effect of *L. paracasei* on DC activation was not limited to *Salmonella* but was also active against immunogenic *Lactobacilli* (not shown), suggesting that *L. paracasei* may also inhibit the activation of the immune system in response to immunogenic commensal bacteria. This is likely the case as when we pretreated mice for 7 days with the three different *Lactobacilli* before exposure to DSS, only *L. paracasei* was protective. As DCs have been shown to regulate the outcome of DSS colitis [40] it is likely that the probiotic effect observed in vivo is dependent on the capacity of *L. paracasei* to inhibit the inflammatory potential of DCs.

Recently, it has been shown that the logarithmic growth phase of *Lactobacilli* is associated with the induction of anti-inflammatory genes [43]. While all of the bacterial preparations used in the current study were logarithmic, still we observed that *LGG* and *L. plantarum* NCIMB8826 were detrimental as they induced the death of DSS-treated mice, suggesting that these strains are inflammatory independently of their growth phase. A recent report has shown that the ratio between IL-10 and IL-12 production after treatment of human peripheral blood mononuclear cells with probiotics, was predictive of in vivo probiotic activity in the TNBS colitis mouse model [44]. The authors showed that a higher IL-10/IL-12 ratio correlated with better in vivo efficacy. Accordingly, in our study, based on MoDCs, we show a worse DAI in mice pretreated with *plantarum* that had a lower IL-10/IL-12 ratio than the other two strains (*plantarum*: 0,877; *LGG*: 1,222; *paracasei*: 1,176). However, the IL-10/IL-12 ratio would not explain the better outcome of *L. paracasei* versus *LGG* that displayed a similar cytokine ratio. It is important to note that *L. paracasei* was a poor inducer of cytokines (both inflammatory and non-inflammatory) and impacted on the ability of DCs to produce inflammatory cytokines in response to pathogens, suggesting that this could represent a new class of immunomodulatory probiotics that do not act via the induction of tolerogenic responses.

In conclusion, we show that probiotics should be divided into immunostimulatory and immunomodulatory according to their ability to interact with immune and non-immune cells, and their clinical use should be tailored accordingly. For instance, *LGG*, which is immunostimulatory has been shown to be more appropriate in the prevention of nosocomial rotavirus-dependent diarrhea in infants [45] or in decreasing the incidence of atopic dermatitis [46], than as an additive therapy in children with CD [47] or in CD patients after surgery [48]. In contrast *L. paracasei*, which is immunomodulatory, may be used to dampen inflammatory responses and may be recommended to maintain the remission phase in IBD. Hence, each probiotic strain should be characterized for their immune activity before being proposed for clinical applications.

## Materials and Methods

### Mice and Bacterial strains

C57/BL6 mice were purchased from Charles River laboratories. All mice were maintained in microisolator cages in a specific pathogen-free animal facility. All experiments were performed in accordance with the guidelines established in the Principles of Laboratory Animal Care (directive 86/609/EEC) and approved by the Italian Ministry of Health.

S. typhimurium strain SL1344 was provided by G. Dougan (The Wellcome Trust Sanger Institute, UK) and grown in LB medium. *Lactobacilli* strains were: *L. plantarum*, NCIMB8826 WT; *L. paracasei* B21060 (Flortec, Bracco); *L. rhamnosus* GG (Dicoflor 30, Dicofarm). All *Lactobacilli* were grown overnight anaerobically at 37°C in MRS broth (Biokar Diagnostic) without shaking. Bacteria were restarted at a 1∶50 dilution and grown to an OD_600_ = 0.6 when the growth is exponential. Bacterial cultures were plated to count effective CFUs. *L. paracasei* supernatant was obtained after centrifugation of the equivalent amount of CFUs of exponential phase bacteria used for DC treatment. *L. paracasei* supernatant was used either undiluted, or diluted 1∶5, 1∶10 or 1∶100 corresponding to nearly 7%, 1,4%, 0,7%, and 0,07% volume/volume of tissue culture medium, respectively.

### Cells and reagents

DCs were derived from human peripheral blood monocytes selected with anti-CD14 antibodies coupled to magnetic beads (Miltenyi, Bologna, Italy) [49]. CD14^+^ cells were incubated for 6 days in complete medium containing granulocyte-macrophage colony-stimulating factor (GM-CSF, 50 ng/mL; Peprotech) and interleukin-4 (20 ng/mL; Peprotech, Milan, Italy) in order to obtain immature MoDCs.

### Bacterial treatments and assessment of MoDC viability

MoDCs were incubated for 1 h with live logarithmic-phase *Lactobacilli* (*L. plantarum*, *L. paracasei* and *LGG*) or with *Salmonella typhimurium* in medium without antibiotics at a 10∶1 (bacteria∶DC) ratio. Cells were extensively washed and the medium was changed to one containing gentamycin (100 µg/ml). Cells were tested 24 hours later for viability after double staining with FITC-conjugated Annexin V (BD PharMingen, San Diego, CA) and 1.25 µg/ml propidium iodide (Sigma Chemical Co.), and analyzed by flow cytometry. Annexin V/propidium iodide double negative cells are indicative of viable cells.

### Epithelial cell monolayers

Caco-2 cells were seeded in the upper chamber of a transwell filter (Costar 3 µm diameter of pores) for 7–10 days until a trans-epithelial resistance (TER) of 300 Ohm•cm^2^ was achieved.

Epithelial cell monolayers were incubated with bacteria (5×10^7^ CFU/TW) upon the apical surface (top chamber). One hour after incubation, bacteria were washed out and medium was changed with one containing antibiotics (gentamycin 100 µg/mL). Culture supernatants were collected 24 hours later from the bottom chamber (facing the basolateral membrane), filtered through a 0.2 µm filter (Nalgene) and used to activate MoDCs.

MoDCs were incubated for 24 hours in culture supernatant (1∶2) and then treated or not with bacteria (10∶1 bacteria to DC) for 1 h in medium without antibiotics. Subsequently bacteria were washed out and cells were left in culture for an additional 23 h in medium containing gentamycin 100 µg/mL. Analysis of cytokines released by epithelial cells or DCs was carried out by testing culture supernatants.

### MoDC T-cell co-cultures

MoDCs were collected after 24 hours of incubation with the different stimuli and then incubated with purified allogeneic CD4^+^CD45RA^+^ T cells (Miltenyi) in 48-well plates (at a ratio of 10 T cells to 1 DC). To measure T cell proliferation: MoDC-T cell were co-cultured for 72 hours, followed by a 16-hour pulse with 1 µCi [^3^H] thymidine (Amersham, Milan). Cell-associated radioactivity was detected after Cell harvesting (TomTec) on filtermats using a Betaplate Counter (MicroBeta TriLux, PerkinElmer).

To measure cytokine release: After 5 days of MoDC-T cell co-culture, supernatants were collected and directly analyzed for cytokine measurements.

### DSS colitis

6 mice per group were administered intra gastrically (i.g.) once a day for 7 days with 200 µl PBS containing 10^10^ CFUs of bacteria grown as above. Control mice were administered with plain PBS. Mice were then fed with 2% DSS dissolved in the drinking water for 5 days without probiotics, followed by 7 days of plain water and analyzed over time for colitis development. Mice were weighed every day and feces were collected to measure consistency and the presence of blood by HEMOCCULT (BeckmanCoulter, Inc). At day 13 after DSS treatment mice were sacrificed.

### Assessment of disease activity

Body weight was assessed at baseline and every day for the duration of the experiment. Weight change was calculated as percentage change in weight compared with baseline. Animals were monitored clinically for rectal bleeding, diarrhea and general signs of morbidity, including hunched posture and failure to groom. Disease activity index (DAI) is the combined score of weight loss, stool consistency, and bleeding. Scores were defined as follows: body weight loss, 0, no loss; 1, 5%–10%; 2, 10%–15%; 3, 15%–20%; 4, 20%; stool consistency, 0, normal; 2, loose stool; 4, diarrhea; bleeding, 0, no blood; 2, presence of bleeding; and 4, gross bleeding [50]

### Cytokine measurements

IL-6, IL-2, IL-12p40, IL-17, IL-12p70, IL-10, IFN-γ, TNF-α and TGF-β concentrations were determined by commercially available ELISA (R&D systems) or Cytokine bead array (Becton Dickinson). Optical densities were measured on a Bio-Rad Dynatech Laboratories ELISA reader at a wavelength of 450 nm (Hercules, CA, USA). CBA-associated Cytofluorimetry was measured by FACS array (Becton Dickinson). Limit of detection of cytokines by CBA<10 pg/ml (for all of them) and by R&D systems TSLP<5 pg/ml, TGF-β<30 pg/ml, IL-8<30 pg/ml.

### Statistical analysis

Student's paired t test was used to determine the statistical significance of the data. Significance was defined as *, p<0.05; **, p<0.01 (two-tailed test and two-sample equal variance parameters). Statistic calculations were performed by JMP 7 software (SAS Cary).

## Supporting Information

Figure S1 Probiotics induce a similar activation of surface markers on DCs. DCs were incubated or not with the reported live bacterial strains for 1 h in medium without antibiotics, washed and incubated for 23 h in medium with antibiotics. Cells were stained for HLA-DR and CD80 expression and analyzed by FACS. Histograms show surface expression of CD80 (left) or HLA-DR (right) in response to the different bacteria (black histograms). Blue histograms show marker expression in unstimulated cells.
(0.24 MB PPT)

Figure S2 Lactobacilli do not differ in their ability to drive CD4+CD25+Foxp3+ T regulatory cells. DCs were incubated or not with the reported live bacterial strains for 1 h in medium without antibiotics, washed and incubated for 23 h in medium with antibiotics. Cells were washed and incubated with naïve CD4+CD45RA+ cells for 5 days (Ratio 1∶10 DC∶T cells). Cells were collected and analyzed by cytofluorimetry for the expression of CD4, CD25 and intracellular Foxp3. S. typhim., Salmonella typhimurium; L. plant., L. plantarum; L. parac., L. paracasei B21060; LGG, L. rhamnosus GG. One representative of three independent experiments is shown.
(0.09 MB PPT)

Figure S3 L. paracasei induces increased levels of TSLP Caco-2 cells were grown as monolayers in the upper chamber of a transwell filter and incubated with bacteria (5×107 CFU/TW) from the apical surface (top chamber). One hour after incubation, bacteria were washed out and medium was changed with one containing antibiotics (gentamycin 100 µg/mL). Cells were collected 3, 5, 23 and 48 hours later from the bottom chamber. mRNA was isolated and retrotranscribed. Quantitative RT-PCR showing TSLP mRNA expression levels normalized to TBP gene are shown. The bars represent normalized TSLP expression values (TSLP/TBP ratios). One of two independent experiments is shown.
(0.14 MB PPT)
