# Supplementary material for: Comparison of the Immunomodulatory Properties of Three Probiotic Strains of Lactobacilli Using Complex Culture Systems: Prediction for In Vivo Efficacy
Source: PLoS One. 2009 Sep 16;4(9):e7056. doi: 10.1371/journal.pone.0007056 (PMC2738944; doi:10.1371/journal.pone.0007056)
Supplement: Figure S1 — Probiotics induce a similar activation of surface markers on DCs. DCs were incubated or not with the reported live bacterial strains for 1 h in medium without antibiotics, washed and incubated for 23 h in medium with antibiotics. Cells were stained for HLA-DR and CD80 expression and analyzed by FACS. Histograms show surface expression of CD80 (left) or HLA-DR (right) in response to the different bacteria (black histograms). Blue histograms show marker expression in unstimulated cells. (0.24 MB PPT) [file pone.0007056.s001.ppt]

## Slide 1
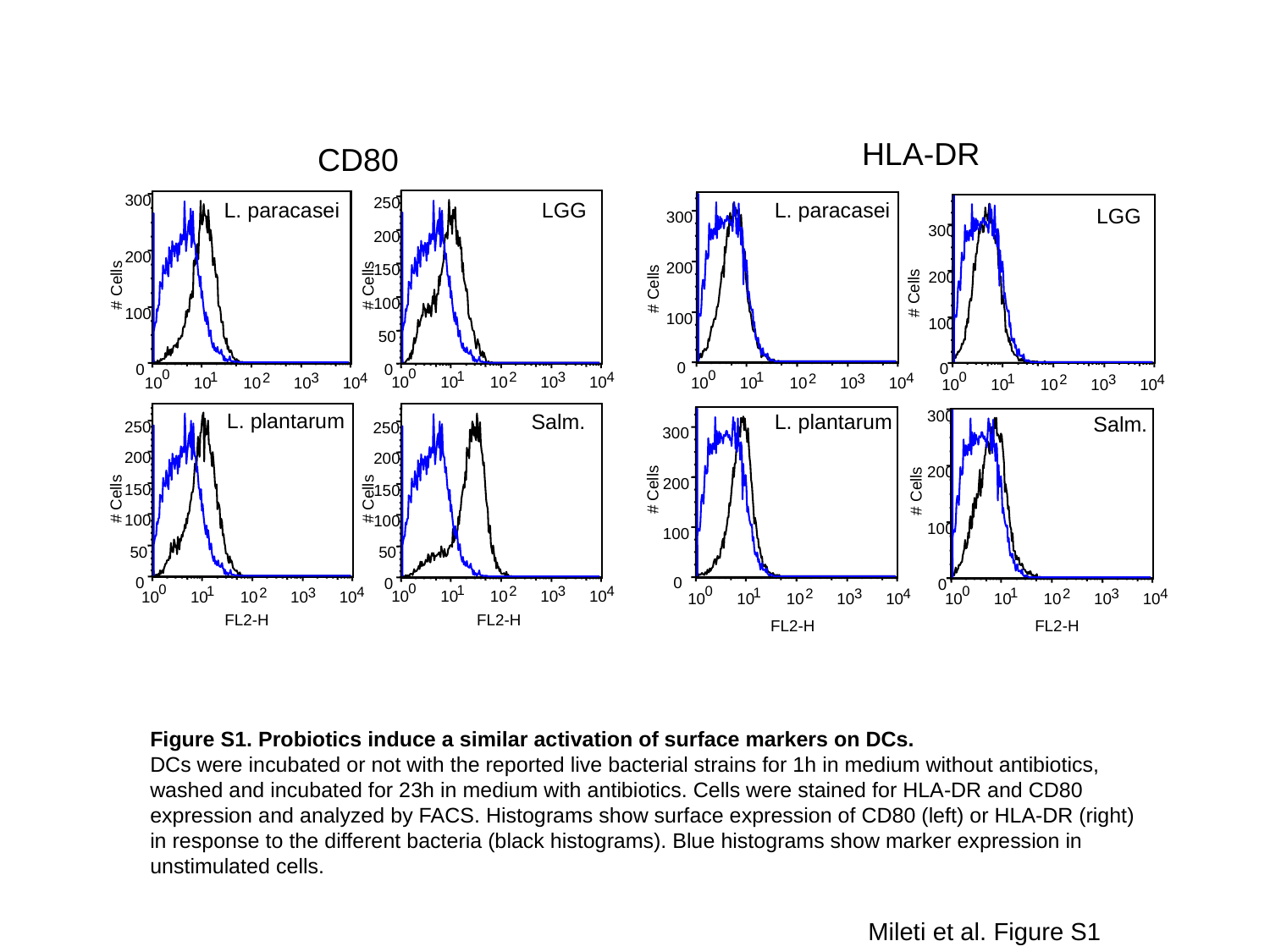

HLA-DR
CD80
300
L. paracasei
LGG
L. paracasei
250
LGG
300
200
100
0
300
200
100
0
200
200
150
# Cells
# Cells
# Cells
# Cells
100
100
50
0
0
0
1
4
2
3
10
10
10
10
10
0
1
4
2
3
10
10
10
10
10
0
1
4
2
3
10
10
10
10
10
0
1
4
2
3
10
10
10
10
10
L. plantarum
Salm.
L. plantarum
Salm.
300
200
100
0
250
250
300
200
100
0
200
200
150
# Cells
150
# Cells
# Cells
# Cells
100
100
50
50
0
0
0
1
4
2
3
10
10
10
10
10
0
1
4
2
3
10
10
10
10
10
0
1
4
2
3
10
10
10
10
10
0
1
4
2
3
10
10
10
10
10
FL2-H
FL2-H
FL2-H
FL2-H
Figure S1. Probiotics induce a similar activation of surface markers on DCs.
DCs were incubated or not with the reported live bacterial strains for 1h in medium without antibiotics, washed and incubated for 23h in medium with antibiotics. Cells were stained for HLA-DR and CD80 expression and analyzed by FACS. Histograms show surface expression of CD80 (left) or HLA-DR (right) in response to the different bacteria (black histograms). Blue histograms show marker expression in unstimulated cells.
Mileti et al. Figure S1
