# Supplementary material for: Comparison of the Immunomodulatory Properties of Three Probiotic Strains of Lactobacilli Using Complex Culture Systems: Prediction for In Vivo Efficacy
Source: PLoS One. 2009 Sep 16;4(9):e7056. doi: 10.1371/journal.pone.0007056 (PMC2738944; doi:10.1371/journal.pone.0007056)
Supplement: Figure S2 — Lactobacilli do not differ in their ability to drive CD4+CD25+Foxp3+ T regulatory cells. DCs were incubated or not with the reported live bacterial strains for 1 h in medium without antibiotics, washed and incubated for 23 h in medium with antibiotics. Cells were washed and incubated with naïve CD4+CD45RA+ cells for 5 days (Ratio 1∶10 DC∶T cells). Cells were collected and analyzed by cytofluorimetry for the expression of CD4, CD25 and intracellular Foxp3. S. typhim., Salmonella typhimurium; L. plant., L. plantarum; L. parac., L. paracasei B21060; LGG, L. rhamnosus GG. One representative of three independent experiments is shown. (0.09 MB PPT) [file pone.0007056.s002.ppt]

## Slide 1
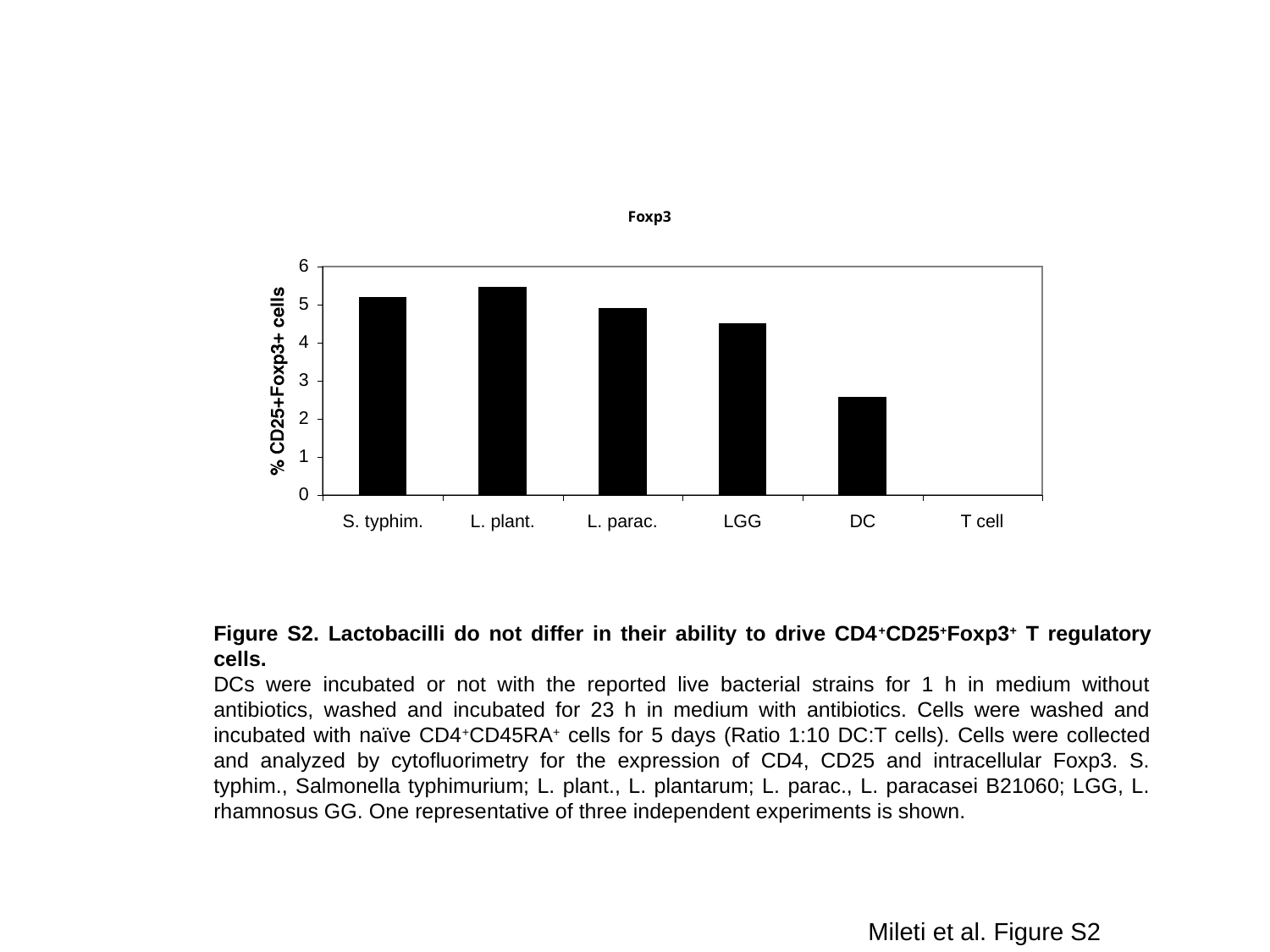

Figure S2. Lactobacilli do not differ in their ability to drive CD4+CD25+Foxp3+ T regulatory cells.
DCs were incubated or not with the reported live bacterial strains for 1 h in medium without antibiotics, washed and incubated for 23 h in medium with antibiotics. Cells were washed and incubated with naïve CD4+CD45RA+ cells for 5 days (Ratio 1:10 DC:T cells). Cells were collected and analyzed by cytofluorimetry for the expression of CD4, CD25 and intracellular Foxp3. S. typhim., Salmonella typhimurium; L. plant., L. plantarum; L. parac., L. paracasei B21060; LGG, L. rhamnosus GG. One representative of three independent experiments is shown.
Mileti et al. Figure S2
