# Supplementary material for: Comparison of the Immunomodulatory Properties of Three Probiotic Strains of Lactobacilli Using Complex Culture Systems: Prediction for In Vivo Efficacy
Source: PLoS One. 2009 Sep 16;4(9):e7056. doi: 10.1371/journal.pone.0007056 (PMC2738944; doi:10.1371/journal.pone.0007056)
Supplement: Figure S3 — L. paracasei induces increased levels of TSLP Caco-2 cells were grown as monolayers in the upper chamber of a transwell filter and incubated with bacteria (5×10^7^ CFU/TW) from the apical surface (top chamber). One hour after incubation, bacteria were washed out and medium was changed with one containing antibiotics (gentamycin 100 µg/mL). Cells were collected 3, 5, 23 and 48 hours later from the bottom chamber. mRNA was isolated and retrotranscribed. Quantitative RT-PCR showing TSLP mRNA expression levels normalized to TBP gene are shown. The bars represent normalized TSLP expression values (TSLP/TBP ratios). One of two independent experiments is shown. (0.14 MB PPT) [file pone.0007056.s003.ppt]

## Slide 1
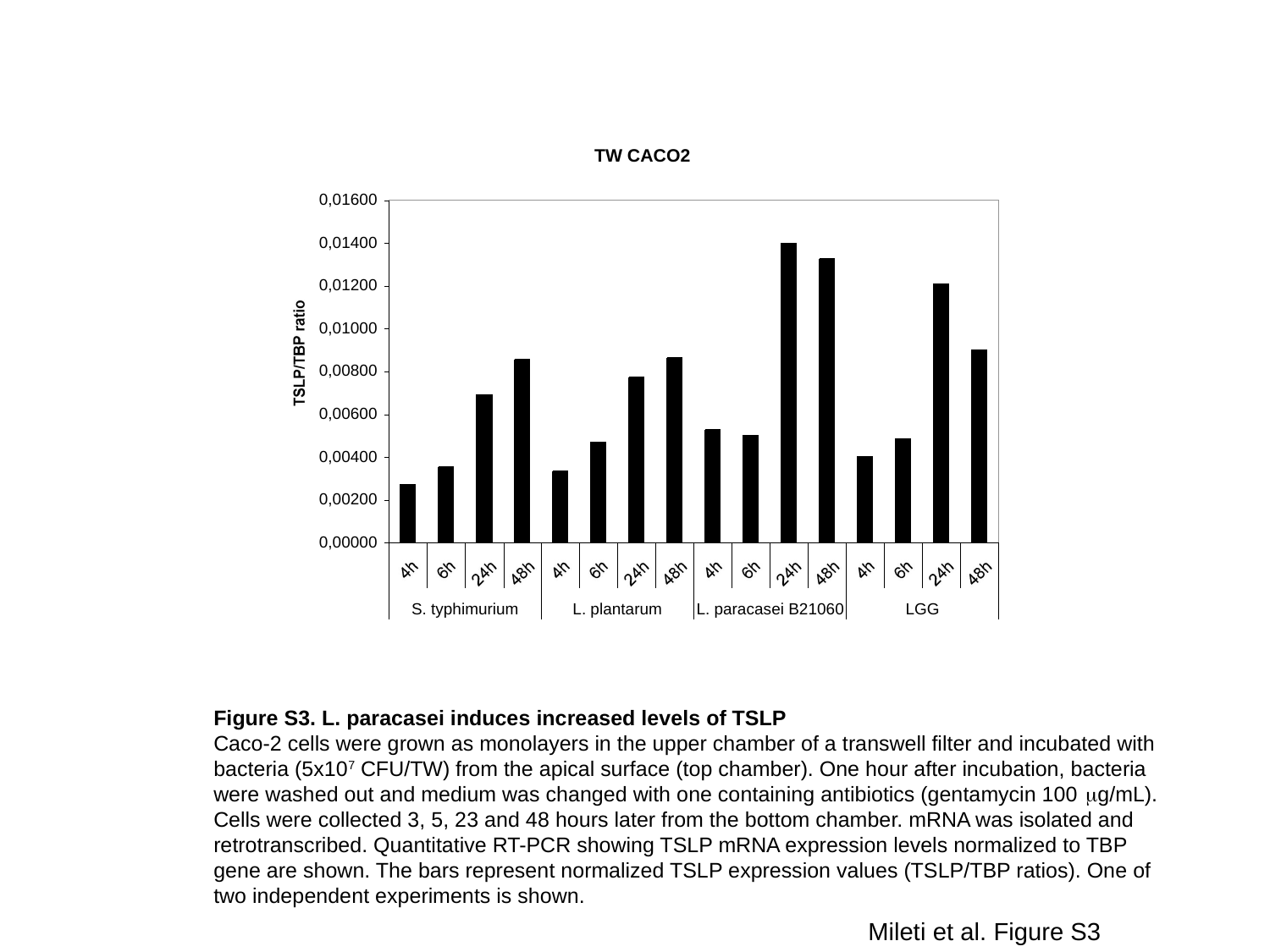

Figure S3. L. paracasei induces increased levels of TSLP
Caco-2 cells were grown as monolayers in the upper chamber of a transwell filter and incubated with bacteria (5x10^7^ CFU/TW) from the apical surface (top chamber). One hour after incubation, bacteria were washed out and medium was changed with one containing antibiotics (gentamycin 100 μg/mL). Cells were collected 3, 5, 23 and 48 hours later from the bottom chamber. mRNA was isolated and retrotranscribed. Quantitative RT-PCR showing TSLP mRNA expression levels normalized to TBP gene are shown. The bars represent normalized TSLP expression values (TSLP/TBP ratios). One of two independent experiments is shown.
Mileti et al. Figure S3
